# Mandibular Chondroblastic Osteosarcoma: A Case Report

**DOI:** 10.7759/cureus.53713

**Published:** 2024-02-06

**Authors:** Yousef M Hasen, Hoda Tawel, Khoulah M Alreeshi, Osama Khalifa, Jamal M Furjani

**Affiliations:** 1 Department of Pathology, Faculty of Dentistry, University of Zawia, Zawia, LBY; 2 Department of Pathology, Faculty of Medicine, University of Zawia, Zawia, LBY; 3 Department of Pathology, Saray Salam Diagnostic Centre, Tripoli, LBY; 4 Department of Oral and Maxillofacial Surgery, New York University College of Dentistry, New York, USA; 5 Department of Oral and Maxillofacial Surgery, Assendan Medical Center, Tripoli, LBY

**Keywords:** mandibular osteosarcoma, chondroid matrix, sun-ray appearance, chondroblastic osteosarcoma, osteosarcoma of the jaw (osj)

## Abstract

Osteosarcoma is primarily a long-bone disease that rarely affects the facial bones. Chondroblastic osteosarcoma is a subvariant of osteosarcoma. Its defining characteristics include the presence of malignant spindle and polygonal cells, as well as a thick layer of chondroid matrix and interwoven neoplastic tissue deposition. Mandibular chondroblastic osteosarcoma, in particular, is often overlooked and disregarded as a presumptive diagnosis at the time of initial presentation. This is mainly because of its rarity or inadequate lesion evaluation. Here, we present the case of a 47-year-old female patient with a rapidly growing swelling at the anterior mandible that was initially misdiagnosed as an ossifying fibroma of the mandible. The subsequent histopathological examination confirmed the diagnosis of chondroblastic osteosarcoma of the mandible. The patient requested a more extensive and aggressive excision, with the possibility of adjuvant radiation or chemotherapy. This article illustrates a rare case of mandibular chondroblastic osteosarcoma, with a focus on the clinical and pathological features of the tumor that should be taken into account when making a differential diagnosis for oral bone lesions.

## Introduction

Osteosarcoma is a primary bone cancer that is distinguished by the presence of the osteoid matrix, which is composed of cancerous cells and deposits on the bone [1]. Osteosarcoma of the jaw (OSJ) is a rare tumor that accounts for 6% to 10% of all osteosarcomas and around 1% of all head and neck malignancies [2]. The mandible and maxilla are the two bones in the craniofacial area that are most frequently affected [2]. Due to its rarity, it may go unnoticed in the differential diagnosis when the patient initially presents.

This disease has a distinct biological behavior from other osteosarcomas of the long bones. It is more frequent in older age groups than younger age groups, with an average age of 10 to 20 years [2]. It also carries more favorable histopathological features, has fewer distant metastases, and has greater survival rates [3]. Most OSJ patients present with jaw swelling as their only symptom; however, the presence of pain, edema, ulcerations, or paresthesia is possible [2]. Radiologically, the tumor may exhibit a range of morphologies, including sclerotic, laminated, and classic sunburst patterns, although it may also occasionally show no discernible radiological abnormalities [4]. To confirm the diagnosis of osteosarcoma when imaging data indicate it, a biopsy is usually necessary.

Histopathologically, osteosarcoma is categorized into classic, telangiectatic, small cell, large cell, spindle cell, periosteal, and parosteal forms. The classical variant of osteosarcoma is further categorized into osteoblastic, chondroblastic, and fibroblastic variants based on the kind of matrix deposited by the neoplastic cells [3]. The chondroblastic type accounts for nearly 25% of all documented cases of osteosarcoma [5]. Chondroblastic osteosarcoma is described as an osteosarcoma with a high level of hyaline cartilage, which is closely connected to the non-chondroid element (i.e., the osteoid or bone matrix generated in the center of the neoplastic cells) [5]. Immunohistochemistry staining can also be a useful diagnostic tool for identifying condroblastic osteosarcoma from other differentials. Immunohistochemistry staining for vimentin, S-100, and galactin-1, for example, might distinguish the disease from chondrosarcoma [6,7].

This case study looks at a chondroblastic osteosarcoma that was initially misdiagnosed as an ossifying fibroma. A second histology investigation utilizing additional samples confirmed the diagnosis. We emphasize that, despite its rarity, OSJ should be considered a differential diagnosis in cases with bony oral lesions.

## Case presentation

A 47-year-old female patient presented with a three-month history of painless, quickly increasing swelling near the anterior mandible. The patient did not reveal a history of comparable past injuries or chronic disease. There was no history of radiation exposure or long-term medication treatment. No documentation existed regarding family history. On examination, the labiomental area was completely damaged with an oral swelling. The lesion was firm and well-defined, measuring around 2 x 2 cm in the symphysis region. The overlying skin was normal, and all anterior teeth tested vital on cold stimuli.

**Figure 1:**
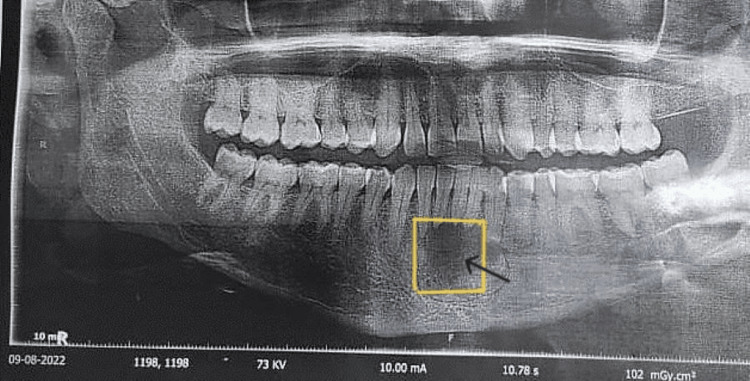
Radiological assessment of the anterior mandibular region. Per-operative panoramic X-ray showed an ill-defined osteolytic lesion in the anterior mandible involving two central incisors and left lateral and canine (low magnification picture) (see the black arrow).

**Figure 2:**
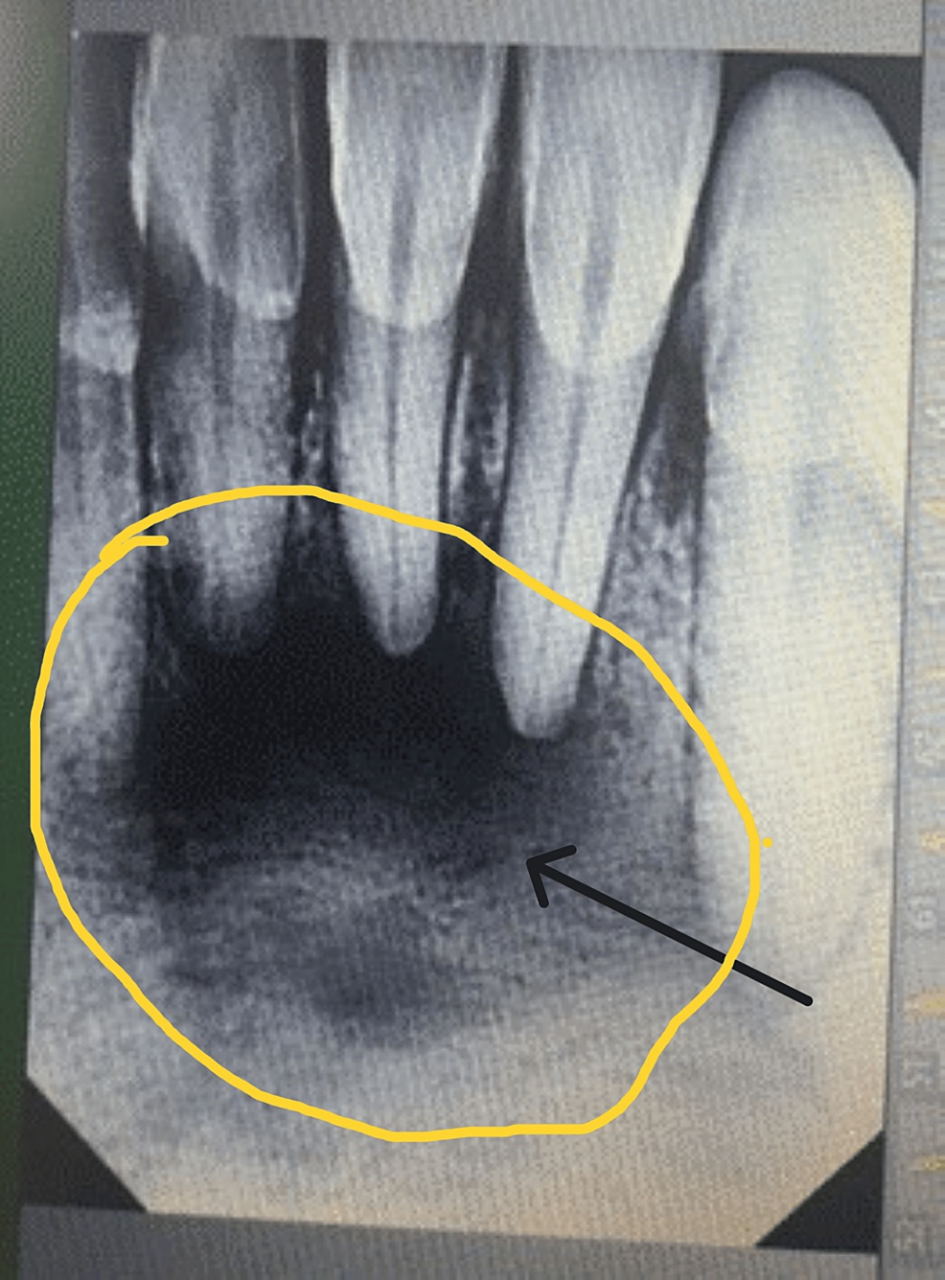
Radiological assessment of the anterior mandibular region. Per-operative panoramic X-ray showing an ill-defined osteolytic lesion in the anterior mandible involving two central incisors and the left lateral and canine (high magnification picture) (see the black arrow).

**Figure 3:**
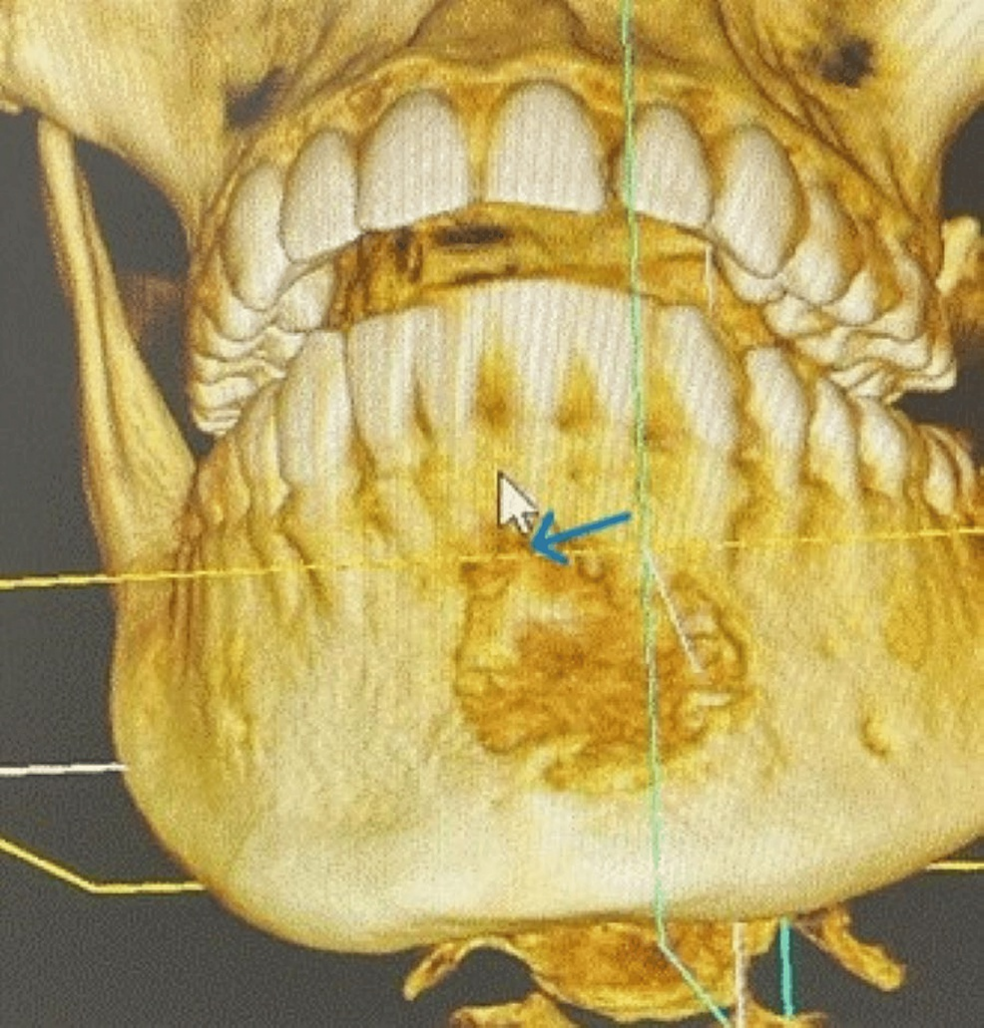
A cone-beam CT scan (before the surgery). An expansile osteolytic lesion with irregular edges and radiopacity in the center at the anterior mandible (see the blue arrow).

The panoramic X-ray image of the jaw revealed that the two central and the left lateral incisors and canine teeth were affected by an undefined osteolytic lesion in the front jaw (Figures 1, 2). In addition, a cone-beam CT scan of the axial cuts revealed an expansile osteolytic lesion with irregular edges and radiopacity in the center (Figure 3). These findings suggested a distractive bony lesion at the mandible. Therefore, an incisional biopsy was performed and sent for histological analysis to determine the underlying cause. As a result, central ossifying fibroma was adopted as the primary diagnosis, as indicated in the referred report. The patient was informed about the treatment plan, and she underwent a local resection with peripheral osteotomy under general anesthesia. The resected sample was sent to the laboratory for further histological analysis and a second opinion.

**Figure 4:**
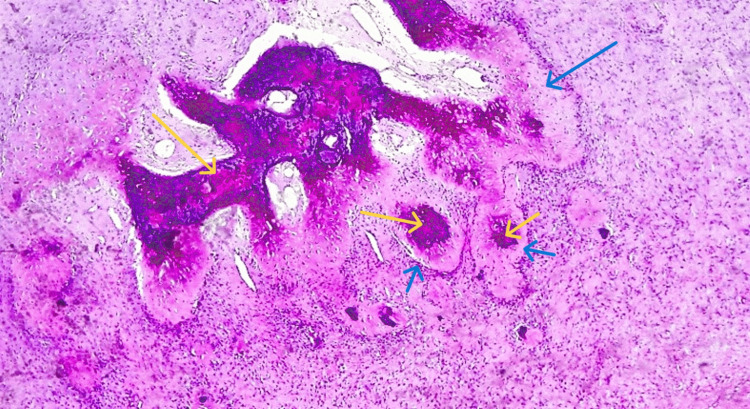
Histopathological assessment of the lesion. Multiple nodules of cartilaginous components (blue arrows) and scattered lace-like disorganized woven bone (yellow arrows), surrounding foci of core bony destruction (microscopic magnification ×10).

Histological evaluation of the resected tissues showed infiltrating tiny, rounded, and spindled neoplastic cells with intramedullary infiltration. Moreover, there were foci of central bony destruction surrounded by multiple nodules of cartilaginous components along with scattered lace-like disorganized woven bone. In addition, evidence of many sites of neoplastic osteoid deposition on native bone (scaffolding) was seen (Figure 4).

**Figure 5:**
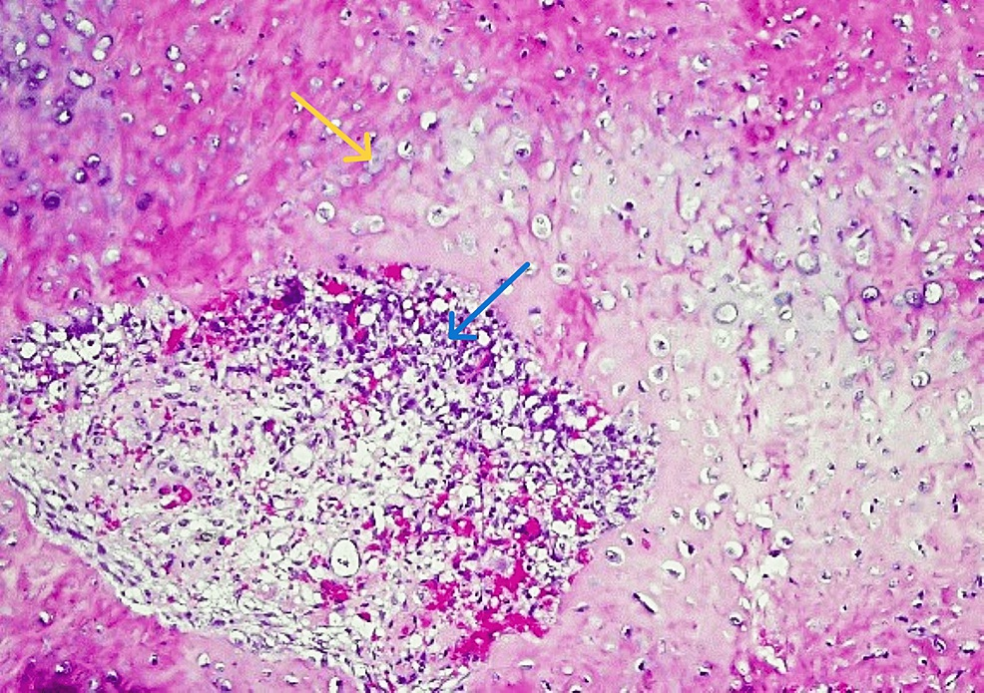
Osteoid production is seen with high-grade malignant cartilage. The tumor biopsy revealing a chondromyxoid matrix (the yellow arrow) and hypercellular neoplasm (the blue arrow).

**Figure 6:**
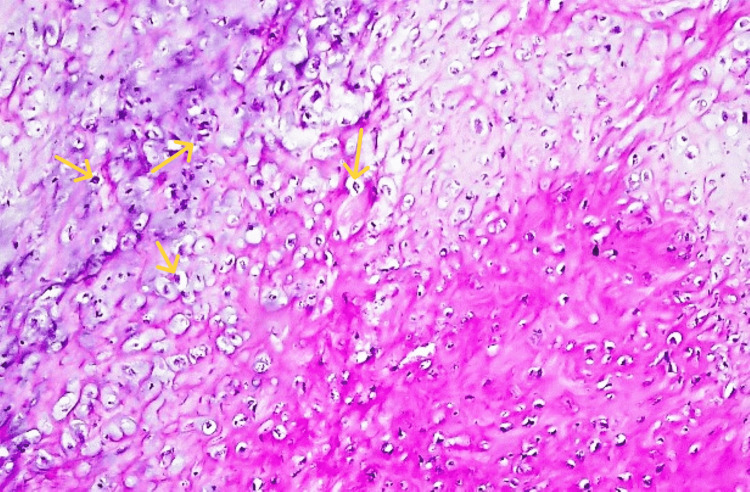
Chondroblastic matrix exhibiting chondrocytes with hyperchromatic and pleomorphic nuclei (yellow arrows).

**Figure 7:**
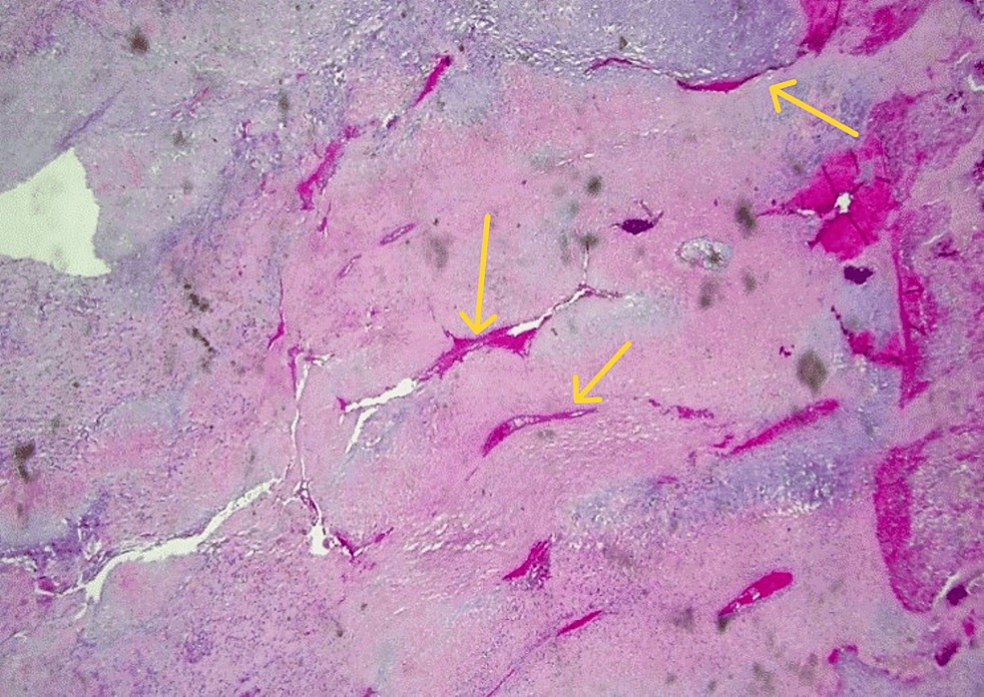
Low-power view of the lesion reveals chondroid tissue with osseous development in the form of bone trabeculae (yellow arrows).

The biopsy also revealed chondromyxoid matrix deposition and hypercellular neoplasm, but no cytological atypia was shown. These findings highly suggested a condroblastic type of osteosarcoma (Figures 5, 6). There was also branching and anastomosing irregularly woven bone trabeculae with osteoblastic rimming and intervening fibrous stroma (Figure 7).

These results demonstrated the existence of a chondroblastic type of OSJ, which required more extensive resection along with adjuvant chemotherapy or radiation therapy. Misdiagnosis in this case, however, was more likely due to the rarity of OSJ and its atypicality.

**Figure 8:**
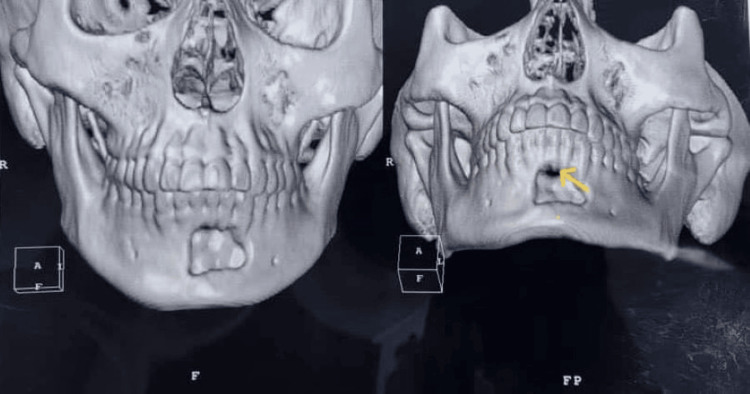
Postoperative CT scan. The scan showed a decrease in the lesion size and new bone deposition (the yellow arrow).

After receiving the final results, the patient was advised that more procedures were needed, such as wide lesion excision and repair, which would be followed by chemotherapy or radiotherapy. However, the patient did not follow through on her follow-up appointments and rejected the treatment plan and any additional surgical intervention. Despite this, the lesion on her most recent CT scan revealed that it had decreased in size and some new bone had deposited at the surgery site after four months of the resection (Figure 8).

## Discussion

OSJ is an uncommon cancer that accounts for about 1% of all cases of head and neck malignancies. In addition, nearly 25% of all reported instances of osteosarcoma are of the chondroblastic type [5]. OSJ is frequently misdiagnosed with other oral disorders such as chondrosarcoma, fibrous dysplasia, or cemento-osseous dysplasia. The rarity and range of histological forms of OSJ, some of which may advance rather subtly, are the primary causes of this [8]. Our patient complained of mandibular swelling, and both radiographic and early histological investigations of the biopsy revealed a primary diagnosis of ossifying fibroma. As a result, the lesion was surgically removed from the patient. Histological examination of the excised tumor, however, confirmed the diagnosis of mandibular chondroblastic osteosarcoma. As a consequence of this, the patient’s treatment regimen was modified. The patient was advised of deep surgical resection, followed by radiotherapy or chemotherapy, as well as further imaging tests to assess the cancer stage.

OSJ might be thought of as a primary tumor with an unknown etiology or often develops as a late complication of radiation therapy to the face, Paget’s disease of the skeleton, or fibrous dysplasia of the bone [9]. In contrast to long-bone osteosarcoma, OSJ occurs one to two decades after puberty, ruling out fast bone development as the primary etiologic reason [9]. However, if OSJ is discovered before the second decade, other osseous abnormalities must be investigated.

The main complaint in our case was rapidly growing mandibular swelling, with no other symptoms such as discomfort, mouth hemorrhage, or oral paresthesia. The majority of documented instances of OSJ had oral swelling. Other signs and symptoms such as oral ulceration, localized discomfort or pain, lip and oral paresthesia, tooth loosening, and/or separation of the teeth are uncommon and are regarded as non-specific manifestations. They are usually associated with the compression of the growing lesion [9]. In extreme cases, nasal obstruction and proptosis have also been observed, indicating central nervous system involvement [9]. Therefore, in situations of mouth swelling, osteosarcoma should be considered as a differential diagnosis.

Based on their location, osteosarcoma may be divided into four categories, namely, extraosseous, periosteal, parosteal, and intramedullary. It may be further subdivided into osteoblastic, chondroblastic, fibroblastic, telangiectatic, and osteoclast-rich forms based on the predominant type of matrix tissue seen under a microscope [4].

CT and MRI are considered essential in the diagnosis of osteosarcoma. They have excellent sensitivity for identifying the morphological alterations produced by tumors [4]. They can also disclose the extent of the tumor’s invasion and depth, as well as its interaction with adjacent tissues or any peripheral calcification [4]. Radiographically, OSJ presents as an osteolytic lesion with periosteal response in 50% of reported cases [10]. The Garrington sign, which is the symmetrical expansion of the periodontal ligament space of the affected teeth on a periapical radiograph, is regarded as an early radiologic indication of osteosarcoma [11]. A sunburst appearance created by radiating mineralized tumor spiculae is seen in some tumors, with most osteosarcomas containing matrix mineralization and calcifications of the osteoid or osteoid-like material inside the tumor [10]. However, these capabilities are not specific to OSJ and can be misleading. Advanced instances frequently involve cortical breakthroughs and disruption of the alveolar border. When it comes to MRI, osteosarcoma exhibits low-to-moderate signal intensity on T1-weighted imaging and high signal intensity on T2-weighted imaging [10].

Histopathological examination of the lesion is regarded as a key method for the diagnosis of OSJ with a high degree of accuracy. OSJ is histologically distinguished by malignant spindle and polygonal cells that produce a neoplastic interwoven osteoid deposition [9]. A chondroblastic subtype of osteosarcoma is proposed if only myxoid regions are present or there is well-developed cartilage. Telangiectatic osteosarcoma, giant cell-rich osteosarcoma, and small cell osteosarcoma can be histologically distinguished by the presence of large blood-filled gaps, giant cell-rich areas, and the presence of small round cells, respectively [9]. Still, some of these types may be misinterpreted as Ewing sarcoma, aneurysmal bone cysts, or giant cell tumors of the bone [8,9]. Our instance showed the existence of malignant cells that are rounded to spindled and have an intramedullary infiltration growth pattern. These cells are linked to the deposition of neoplastic osteoid tissue. In addition, the lesion included a large number of cartilaginous nodules, which ultimately indicated the presence of a condroblastic form of OSJ.

It is difficult to accurately diagnose condroblastic osteosarcoma in the jaw using small tissue sampling techniques such as incisional biopsy or fine-needle aspiration. If osteoid tissue is confused with a chondroid matrix, an osteosarcoma might be mistaken for a chondrosarcoma [5]. With a small tissue sample, it might not be feasible to distinguish between condroblastic osteosarcoma and dedifferentiated chondrosarcoma with osteosarcoma as the sarcomatous component [5]. For instance, a case of misdiagnosed sacral chondroblastic osteosarcoma has been reported. The first cytopathologic diagnosis was chordoma in fine-needle aspiration cytology, chondrosarcoma in incisional biopsy, and condroblastic osteosarcoma in total resection material [12]. Therefore, additional caution is required for the diagnosis of OSJ.

Immunohistochemistry staining could be also to identify condroblastic osteosarcoma from other differentials, such as chondrosarcoma [7]. For instance, S-100 and vimentin are both expressed positively in chondrosarcomas but not in osteosarcomas [13]. Nevertheless, S-100 may be focally positive in the cytoplasm of cartilaginous cells in some instances of condroblastic osteosarcoma [9]. Other immunological markers as galactin-1 can also be used as a diagnostic marker for osteosarcoma, as it is constantly found to be positively expressed. Galactin-1 is highly expressed in osteosarcoma but was not found in chondrosarcoma, with an 85.7% positive predictive value [6]. Furthermore, the proliferation marker MIB-1, which recognizes the Ki-67 antigen, can be utilized to offer independent prognostic information for osteosarcoma [9].

Age at diagnosis, histologic subtype, tumor grade, tumor size, presentation stage, surgery, and radiation therapy have been demonstrated to be prognostic factors and drivers of survival in patients with OSJ [3,14]. Advanced age, high-grade tumors, tumor size, and a higher presentation stage are all considered independent predictors of poor survival [3]. Nevertheless, there is no discernible difference in OSJ patients’ survival based on their ethnicity, gender, or main location [3,14]. Histologically, it has been shown that the chondroblastic, fibroblastic, and parosteal subtypes of the illness have excellent prognoses, whereas the osteoblastic subtype has a less favorable outcome [3]. Moreover, the presence of additional disorders, such as Paget disease, also carries a bad prognosis [3,14]. On the other hand, it has been highlighted that radiation treatment and surgical excision both enhance survival [3].

The cornerstone of treatment for primary jaw osteosarcomas is adequate surgical resection. In more severe cases, palliative treatments such as chemotherapy or radiation may be given either alone as a palliative treatment, or in conjunction with surgical resection. According to the literature, individuals who first had hemi-mandibulectomy or other severe local or even radical surgery recovered better [11]. Radiation therapy for the treatment of the disease is still debatable and hardly acknowledged. Nevertheless, it should be taken into account if there are positive margins, high-grade tumors, the tumor cannot be surgically removed, or the patient has relapsed [15].

Our patient needed further radical surgical resection followed by radiotherapy or chemotherapy, along with additional imaging tests for cancer staging. Although condroblastic osteosarcoma has a favorable prognosis, in our situation, it was impossible to assess the prognosis because the patient declined further treatment or monitoring. Therefore, there is a significant chance that the tumor in our patient might advance and return.

## Conclusions

It is crucial to take into account both the size of the primary tumor and the presence of any distant metastases when making a diagnosis of OSJ. The initial OSJ diagnostic evaluation should include a thorough medical history, a physical examination, and a radiographic assessment of the afflicted jaw and chest. OSJ, despite its rarity, should be one of the differential diagnoses in cases with bony oral lesions.
